# Computer-based test (CBT) and OSCE scores predict residency matching and National Board assessment results in Japan

**DOI:** 10.1186/s12909-021-02520-2

**Published:** 2021-02-02

**Authors:** Shoko Horita, Yoon-Soo Park, Daisuke Son, Masato Eto

**Affiliations:** 1 Office for Clinical Practice and Medical Education, Graduate School of Medicine, The University of Tokyo, Tokyo, Japan; 2 Massachusetts General Hospital, Harvard Medical School, Boston, MA USA; 3 International Research Center for Medical Education, Graduate School of Medicine, The University of Tokyo, Tokyo, Japan; 4 Department of Community-based Family Medicine, School of Medicine, Tottori University Faculty of Medicine, Yonago, Japan

## Abstract

**Context:**

The Japan Residency Matching Program (JRMP) launched in 2003 and is now a significant event for graduating medical students and postgraduate residency hospitals. The environment surrounding JRMP changed due to Japanese health policy, resulting in an increase in the number of unsuccessfully-matched students in the JRMP. Beyond policy issues, we suspected there were also common characteristics among the students who do not get a match with residency hospitals.

**Methods:**

In total 237 out of 321 students at The University of Tokyo Faculty of Medicine graduates from 2018 to 2020 participated in the study. The students answered to the questionnaire and gave written consent for using their personal information including the JRMP placement, scores of the pre-clinical clerkship (CC) Objective Structured Clinical Examinations (OSCE), the Computer-Based Test (CBT), the National Board Examination (NBE), and domestic scores for this study. The collected data were statistically analyzed.

**Results:**

The JRMP placements were correlated with some of the pre-CC OSCE factors/stations and/or total scores/global scores. Above all, the result of neurological examination station had most significant correlation between the JRMP placements. On the other hand, the CBT result had no correlation with the JRMP results. The CBT results had significant correlation between the NBE results.

**Conclusions:**

Our data suggest that the pre-clinical clerkship OSCE score and the CBT score, both undertaken before the clinical clerkship, predict important outcomes including the JRMP and the NBE. These results also suggest that the educational resources should be intensively put on those who did not make good scores in the pre-clinical clerkship OSCE and the CBT to avoid the failure in the JRMP and the NBE.

## Introduction

Objective structured clinical examinations (OSCE) [1] have been used worldwide for decades and are currently used to assess medical students in more than 100 countries. In Japan, all medical schools and faculties of medicine in universities use a pre-clinical clerkship (CC) OSCE [2] as one of examinations for promotion to CC together with the Computer-Based Test (CBT), which is another assessment of medical knowledge applied before CC.

Following worldwide trends to assess clinical competence just before graduation, the post-CC OSCE has just started in almost all medical schools in Japan as an examination for graduation. Apart from the post-CC OSCE, graduates must pass the national board examination (NBE), which in Japan is based on a marksheet test, evaluating only medical knowledge. Doctor of Medicine is awarded to those who completed a 6-year course in a university faculty of medicine or medical college after high school graduation in Japan.

In most cases medical students take the pre-CC OSCE and CBT in their fourth grade, and usually they need to pass both assessments to proceed to CC. The pre-CC OSCE is provided by a Public Interest Incorporated Association, Common Achievement Tests Organization (CATO) and includes nine stations: (1) medical interview; (2) basic life support; (3) basic medical procedure; physical examinations of (4) head and neck, (5) chest, (6) vital signs, (7) abdomen, and (8) limb and spines; and (9) neurological examinations. Among these stations, (1), (2) or (3), (4), (5) or (6), (7), and (9) are compulsory. The CBT is also provided by CATO. It consists of 248 questions, randomly selected for individuals from about thirty thousand of pooled questions. On the other hand, the NBE is provided by The Ministry of Health, Labour, and Welfare. Medical students in their last year and those who have graduated from medical school or a university faculty of medicine take the NBE to get their doctor’s license. This examination is administered over 2 days, as marksheets with 400 questions. A hundred questions are “essential,” requiring at least 80% correct for qualification. In Spring 2020, 10,462 students applied for the examination and 9341 of them passed it.

In the University of Tokyo (UTokyo) Faculty of Medicine, the medical students take general academic subjects in the first one and half years. After that the students take basic medical science such as biochemistry, physiology for one and half year, until the end of the third grade. In the fourth-grade students take clinical medicine and physical examination. They proceed to CC after the success of Pre-CC OSCE and CBT. In the UTokyo, the pre-CC OSCE has six stations: (1) medical interview; either (2) basic life support or (3) basic medical procedure; physical examinations of (4) head and neck, either (5) chest or (6) vital signs, (7) abdomen; and (9) neurological examinations (Table 1). Checklists assessing concern for patients (about 10 checklists) and the examination process (about 20 checklists) are scored point by point and a global score (GS, best = 6, worst = 1) are calculated for each OSCE. A “pass” is given to those who get 60% or higher on the checklists and 3.0 or higher on the GS. The students also take the post-CC OSCE, which is uniquely produced by the UTokyo, just before graduation, a process that started in 2016. There are several stations; two or three clinical scenarios (randomly assigned from six scenarios) and two basic procedures. Checklists of clinical reasoning and GS (best = 6, worst = 1) are used to score the post-CC OSCE. Students with 3.0 and higher at each station qualify for graduation.

**Table 1:** Pre-clinical clerkship (CC) Objective structured clinical examinations (OSCE)—in The Tokyo Faculty of Medicine, a pre-CC OSCE was performed as below, in the fourth grade, in autumn. In total, the students took six stations in the examination

	Assessment Factors	Concerns/Processes	Skills/Contents	Total Score	Global Score
Medical Interview		N/A	N/A	○	○
Physical Examinations	Head & Neck	○	○	○	○
	Chest^a^	○	○	○	○
	Vital^a^	○	○	○	○
	Abdomen	○	○	○	○
	Neurological examinations	○	○	○	○
Basic Life Support		N/A	N/A	○	○

^a^ either selected yearly

So far, the NBE has been well studied and shown to have a significant correlation with CBT [3]. In contrast, although there is a vast body of literature about the method of OSCE itself–its merits, drawbacks, and limitations [4–6] –current studies on the predictive validity of OSCE, both pre-CC and post-CC, are not sufficient [7]. And as such, using Messick’s unified validity framework [8] as the conceptual theory supporting the pre-CC and post-CC OSCEs, we examine validity evidence targeting relations to other variables and consequences that address predictive association. Gathering validity evidence relating to these predictive aspects of the pre-CC and post-CC OSCEs would support the types of inference and use made with their scores, motivating the need for this study.

On the other hand, the residency matching program in Japan [9] started in 2003. The Japan Residency Matching Program (JRMP) provides both medical students expecting graduation or graduates and training hospitals a matching system. In this system, the applicants for resident (students expecting graduation or graduates) and training hospitals are optimally matched by a specific computed algorithm. The training hospital recruit medical students in the last year or graduates according to their original criteria, usually by the score of paper tests like the NBE and/or an essay, and interviews. In Autumn 2019, 10,075 students and 1020 training hospitals (1363 training programs) joined this program. With the algorithm, 9042 students matched a training hospital, while 742 students did not get a match and had to look for training hospitals with vacant trainee positions by themselves.

About 90–95% of the applicants are matched every year and applicants without a match must find training hospitals by themselves. So, far, the ratio of hospital capacity to applicants has been about 1.2, however the Ministry of Health, Labour and Welfare aims to lower the ratio to 1.05. For hospitals in metropolitan areas, the capacity will be compressed more than ever than before in order to distribute training doctors to rural regions. Because of this we expect the number of students or graduates with unsuccessful matches will increase soon.

The result of matching between medical students and a residency program is regarded as an important educational outcome [10, 11]. Chakraborti and colleague tried to assess the educational quality of medical training quantitatively by using a match quality score [10]. Chang et al. developed a novel evaluation method of undergraduate medical education [11]. These results suggest that the success of matching medical students and residency hospitals is regarded as an important educational outcome. Medical schools will be obliged to analyze the result of the matching and develop programs to avoid unsuccessful matches.

Needless to say, an unsuccessful match is a big burden for students. Unsuccessful matches are beginning to be a concern in North America as well; however, there are a few studies with statistics analyzing unsuccessful matches and establishing educational strategy [12], nor the studies investigating the factors involved in unsuccessful matches [13]. Studies show that in Canada students apply for twenty residency programs in average until they are matched [14, 15].

Moreover, although not many literatures pointing out that daily life such as meals, sleep, self-studying time, part-time jobs and club activities, correlates with examination marks, there are some international and domestic literatures that point out that lack of breakfast and sleep disorder are significantly correlated with low examination results [16, 17].

The predictive relationships between pre-CC and post-CC OSCEs with JRMP and NBE results would provide meaningful information on their use. Our work hypothesizes relationships between the pre-CC OSCE result and JRMP results, and between the post-CC OSCE and the NBE results. Moreover, we also suspected that daily life may influence their scores and these important educational outcomes. To prove these hypotheses, we planned this investigation.

## Methods

Ethical approval No.11637 was given by the ethical committee of The UTokyo Faculty of Medicine. All the methods were performed in accordance with the relevant guidelines and regulations.

### Sample population

Students at The UTokyo Faculty of Medicine answered a questionnaire about daily activities such as commuting hours, meals, sleep, self-study hours and part-time job hours after giving their written consent to participate. Their scores on official domestic examinations (pre- and post-CC OSCEs and CBT) and related personal data were collected with their written consents and kept in the university affairs system, with permission of the ethical committee of The UTokyo (No. 11637). In total, 237 out of 321 students participated in this study. The fundamental data, including information about 2018, 2019, and 2020 graduates, are shown in Table 2, Tables 3, and 4.

**Table 2:** Important educational outcomes of The University of Tokyo Faculty of Medicine

Japan Residency Matching Program (JRMP) and national board examination (NBE) results				
Graduation year		2018	2019	2020
Number of graduates	*1	106	115	100
NBE pass	*1	98	106	96
NBE fail	*1	8	9	4
JRMP match	*2	53	72	68
JRMP unmatch	*2	5	8	0
JRMP unknown	*2	11	5	9
NBE pass ratio	*1	92.5	92.2	96.0

*1: officially announced

*2: students with consents only

**Table 3:** Comparison of important outcome statistics among the 2018, 2019, and 2020 graduates

Graduation year	2018 (***n*** = 69)	2019 (***n*** = 85)	2020 (***n*** = 83)	Tests
CBT score (%)	81.1 ± 1.12	77.4 ± 1.25	80.8 ± 1.10	Tukey–Kramer *p* > 0.05
CBT-IRT	549 ± 14.1	505 ± 13.0	546 ± 11.8	Tukey–Kramer *p* > 0.05
Pre-CC OSCE total score by factor (%)	86.5 ± 0.662	86.9 ± 0.594	87.7 ± 0.432	Steel–Dwass *p* > 0.05
Pre-CC OSCE global score (6 full)	4.50 ± 0.0559	4.42 ± 0.0470	4.43 ± 0.0437	Tukey–Kramer *p* > 0.05
NBE pass ratio (%)	94.2	91.0	96.4	Chi Fisher test *p* > 0.05

Mean ± SD

**Table 4:** Comparison of JRMP match ratio

Graduation	2018 (***n*** = 58)	2019 (***n*** = 80)	2020 (***n*** = 74)	Chi Fisher test
JRMP match ratio (%)	91.4	90.0	91.0	*P* > 0.05

### Examinations

Table 1 shows the evaluation factors of the pre-CC OSCE at The Tokyo. The pre-CC OSCE was administered as below, with six stations, in the autumn of fourth year.

The CBT was performed by CATO, again in the autumn of fourth year at The Tokyo. Each student was given 248 questions randomly selected from a large pool of questions.

The post-CC OSCE was administered as a graduation examination for sixth-year students, in early winter. Each student completed six examinations of clinical scenarios and two skill performance examinations.

### Data analysis

The answers to the questionnaires, the examination scores (pre- and post-CC OSCEs, CBT, the JRMP results and the NBE) were analyzed with JMP version 14.0 (SAS Institute, N.C., USA) and SPSS version 26 (IBM Corp., Armonk, N.Y., USA) software. The statistic method used for each analysis depended on the characteristics of the population. The cut-off for the odds ratio was determined at the maximum area under the receiver operating characteristic curve.

## Results

### Comparison with Japan residency matching program (JRMP) unmatched versus matched

We first analyzed two-group comparison of each factor (station) of the pre-CC OSCE between students who were successfully matched through JRMP and those who were unmatched. As shown in Table 5, some of the pre-CC OSCE factors (stations) and/or total scores/GS had significant relationships with JRMP results. The elements that evaluate sympathy and communications with patients had stronger relationships with JRMP results of being matched or unmatched. On the other hand, the elements that evaluate skills and knowledge did not have a significant relationship between JRMP results of being matched or unmatched.

**Table 5:** Pre-CC OSCE results bv contents vs. JRMP results—positive t-test

OSCE factors/station	JRMP Match (mean ± SE)	JRMP Unmatch (mean ± SE)	***P***-value	cut-off	Odds ratio	95% CI
Neuro_Score_CP	93.3 ± 0.681	80.5 ± 4.44	0.0003^*1^	92.6	7.07	1.99–25.1
Total_Score_CP	95.0 ± 0.369	89.5 ± 2.33	0.0075^*1^	91.1	5.02	1.90–13.3
Neuro_Score_Sum	86.2 ± 0.668	79.3 ± 2.96	0.0134^*1^	88.3	4.38	1.24–15.5
Total_GS	4.46 ± 0.0304	4.22 ± 0.103	0.0205^*1^	4.58	3.70	1.04–13.2
Total_Score_Sum	87.2 ± 0.359	84.5 ± 1.18	0.0265^*2^	90.1	8.52	1.11–65.3
Neuro_GS	4.50 ± 0.0562	4.10 ± 0.193	0.0323^*2^	4.00	(2.40)^*3^	0.79–7.24
CBT-IRT (as a reference)	535 ± 8.36	490 ± 22.1	0.1071	–	–	–

Abbreviations: *CP* Concerns and process, *NE* Neurological examinations, *HN* Head and neck, *GS* Global scores, *SC* Skills and contents, *VS* Vital signs, *HN* Head and neck

*1: Wilcoxon/Steel–Dwass test

*2: Student/Tukey–Kramer test

*3: *p* > 0.05 on Fisher’s test

Among the six stations (medical interview, head and neck, chest/vital sings, abdomen, neural examination, and basic life support), results of the neurological examination station had significant correlation with the JRMP result (Table 5). Additionally, the “concerns and process” (CP) factor, which evaluates the consideration for the patients, also had significant correlation with the JRMP result (*p* = 0.0075). The neurological examination station had significant correlation with JRMP results, GS, total score, and CP factor score (*p* = 0.0323, 0.0134, and 0.0003). This characteristic was not seen in any other stations. The whole pre-CC OSCE score (total score and GS) also had strong correlation with JRMP results (*p* = 0.0265 and 0.0205). On the other hand, stations for examining vital signs, abdomen, chest, and the “skills and contents” (SC) factor did not have significant correlation with JRMP results. Additionally, the CBT score did not have a correlation between JRMP placement being matched or unmatched.

We also checked the relationship between the background of the students and JRMP results. The students who graduated public (not national) high school had lower JRMP placement rates than the students who graduated private or national high school (odds ratio 4.542). Importantly, students who were promoted to medical school through a separate screening process after admission received significantly higher scores and JRMP placement rates than students who were directly admitted to medical school (odds ratio 1.6762). Daily activities such as commuting hours, meals, sleep, self-study hours and part-time job hours did not have a significant correlation between JRMP results (data not shown).

### Comparison with national board examination (NBE) pass or fail

We investigated the correlation between CBT, pre-CC OSCE scores, and NBE results (Table 6). In contrast to the correlations between these scores and JRMP results, CBT scores had significant correlation with NBE results (*p* < 0.0001, odds ratio infinite). Also, in contrast to JRMP placement, as for pre-CC OSCE results, the total SC factor, which did not have significant correlation between JRMP placements, had remarkable correlation with NBE results. Other factors such as total score, total GS, the total CP factor, head and neck examination and vital examination stations of the pre-CC OSCE also had significant correlation with NBE results. The daily activities, commuting hours, meals, sleep, self-studying hours and part-time job hours, did not have significant correlation with NBE results (data not shown). However, those who did not pass the NBE had a higher frequency of not having a morning meal. Surprisingly, the repetition did not have a correlation with NBE placement (data not shown).

**Table 6:** CBT/Pre-CC OSCE results vs. NBE results: positive T-test

	NBE pass (mean ± SD)	NBE fail (mean ± SD)	***P***-value	cut-off	Odds ratio	95% CI
CBT scores: %	80.8 ± 0.644	61.7 ± 2.57	< 0.0001^*2^	74.19	∞	
CBT-IRT	545 ± 7.23	357 ± 17.0	< 0.0001^*1^	433.0	∞	
OSCE Total_Score_Sum	87.3 ± 0.318	83.2 ± 1.92	0.0261^*1^	83.69	5.27	1.74–16.0
OSCE Total_GS	4.46 ± 0.0286	4.22 ± 0.114	0.0401^*2^	4.583	4.13	0.90–18.9
OSCE Total_Score_SC	85.7 ± 0.438	80.2 ± 2.87	0.0628^*1^	81.05	4.73	1.57–14.3
OSCE Total_Score_CP	94.9 ± 0.371	91.3 ± 2.08	0.0318^*1^	92.86	4.19	1.53–13.0
OSCE Vital_Score_CP	94.3 ± 0.671	89.3 ± 2.86	0.0873^*2^	(85.71)	4.58	1.07–19.5
OSCE Vital_Score_SC				87.50	(6.35)	0.76–53.0
OSCE Vital_Score_Sum				66.67	11.83	2.34–59.8
OSCE HN_Score_SC	89.6 ± 0.605	83.9 ± 2.41	0.0242^*2^	90.00	4.35	1.18–16.0
OSCE HN_Score_Sum	91.3 ± 0.486	86.5 ± 1.94	0.0164^*2^	90.74	4.47	1.36–14.7
OSCE Neuro_Score_Sum	86.2 ± 0.646	80.9 ± 2.58	0.0497^*2^	(76.67)	4.83	1.56–14.9

Abbreviations: CP, concerns and process; NE, neurological examinations; HN, head and neck; GS, global scores; SC, skills and contents; VS, vital signs; HN, head and neck

*1: Wilcoxon/Steel–Dwass test

*2: Student/Tukey–Kramer test

### Comparison between domestic tests, JRMP placement, and NBE results

The result of the domestic post-CC OSCE had strong correlation with NBE results (*p* < 0.0001) (Table 7). Compared to the whole post-CC OSCE that included skills tests, the post-CC OSCE with clinical scenarios had a higher correlation with NBE results. On the other hand, the correlation of post-CC OSCE between JRMP matched or unmatched was not as high as between CBT. The global scores of CCs in the sixth year also had a relationship between JRMP and NBE results, however, the global scores of CCs in the fifth year had no relationship between them (data not shown).

**Table 7:** Post-CC OSCE results vs. JRMP/NBE results

	JRMP (***n*** = 212)			NBE (***n*** = 237)		
	Match (mean ± SD)	Unmatch (mean ± SD)	***p***-value*	Pass (mean ± SD)	Fail (mean ± SD)	***p***-value*
Post-CC OSCE (clinical) GS	4.40 ± 0.0500	4.13 ± 0.167	0.140	4.46 ± 0.0429	3.27 ± 0.155	< 0.0001
Post-CC OSCE (clinical & skills) GS	4.39 ± 0.0606	4.06 ± 0.139	0.0344	4.41 ± 0.0386	3.56 ± 0.385	0.0002

* Student’s t-test

### Comparison between daily life and pre-CC OSCE, CBT, JRMP placement, post-CC OSCE and NBE results

We also analyzed whether daily life factors, such as daily activities such as commuting hours, meals, sleep, self-study hours and part-time job hours, are correlated with test scores as above. In this study we did not find significant correlation between these daily life factors and the scores of pre-CC OSCE, CBT, JRMP, post-CC OSCE and NBE results.

## Discussion

### Main findings and interpretation

The most important finding of this investigation is that the placement through JRMP, an established Japanese residency matching, has significant correlation with the validations of the pre-CC OSCE, thereby supporting the validity evidence of the assessment.

The JRMP launched in 2003. This system has changed the environment of Japanese residency, from university-hospital-based residency to predominantly community hospitals, presumably because in the community hospitals residents can practice more than in university hospitals. However, it has a systematic defect; as many medical students can freely select a hospital unrelated to their university, the top community hospitals, most of which are located in the metropolitan and urban areas, collect a lot of excellent students. As a result, hospitals in the areas suffer from lack of residents. On the other hand, as these top community hospitals have higher rates for application than the other hospitals, some students are unsuccessful in the JRMP. As The Ministry of Health, Labour and Welfare intends to limit the residency in the metropolitan and urban areas, the number of students who are unsuccessful at getting a match through JRMP will certainly increase in the near future [18].

It is rather surprising that the scores of the pre-CC OSCE and JRMP placements have significant correlation with each other and that it depends on the stations and evaluation items of the pre-CC OSCE. The points are 1) the CP evaluation item has higher correlation with the JRMP result than SC, and 2) the scores of the neurological examination, and head and neck stations have higher correlation between JRMP results than the other stations, in almost all items of the evaluation. On the other hand, the SC of the pre-CC OSCE did not have significant correlation with the JRMP results. Additionally, scores of some stations such as abdomen examination and vital examination did not have significant correlation between the JRMP results.

The result that the score of the neurological examination station significantly correlated to JRMP also suggests that the quality of examiners is excellent. In the pre-CC OSCE of The UTokyo the examiners for each station are selected by consulting each division; neurologists, neurosurgeons, and orthopedists usually take charge of the evaluation of neurological examination station. There is discussion about the training of examiners: some argue that training the examiners is not useful in raising the reliability of the evaluation [19, 20], while others argue that training the examiners ahead of the examination is useful [21]. It is not appropriate to decide which is exactly correct, but we could say that the basic clinical knowledge, skills, and experiences as an examiner will contribute to raise the quality of the evaluation of OSCE. This also suggests that the questionnaire of the neurological examination is well designed for evaluating not only the skills of neurological examination but also communication skills as well. This is due to the questionnaire at the neurological examination station being more difficult and complicated than the other stations In the detail, the neurological examination contains examinations around the face of the patients, such as cranial nerves, which needs more careful attention and more sufficient explanation to the patient.

There have been no published studies about the correlation between JRMP placement and scores, including official high-stakes examinations and daily evaluations of CCs of each medical school. The recruitment criteria of the residency hospitals, including university hospitals and other residency hospitals, depend on each institution. We estimate this reason as below: 1) the evaluation items of the CP factor of the pre-CC OSCE well evaluate the communication skills of each student to some extent, and 2) in their recruitment of residents, the residency hospitals consider communication skills to be of great importance.

In this study we did not find significant correlation between daily life and examination/JRMP results. However, we found some tendency that too much part-time job hours and too much club activities lower the NBE result (data not shown). To clarify this, we need more participants into this study.

In general, the recruitment examinations of Japanese residency hospitals include paper tests on medical knowledge and/or an essay, and face-to face interviews, depending on each institution. These tests evaluate the medical knowledge, the communication skills and sometimes medical skills of each applicant. Whether performed face-to-face or by video, an interview is inevitable for evaluating professionalism, interpersonal, and communication skills [22–24]. In this viewpoint, the fact that the JRMP placement and the NBE placement do not correlate with each other is very intriguing and may be related to the situation in other countries [25]. In 2007, Peskun and Shandling have reported that in Canada, residency ranking in internal medicine and family medicine is significantly correlated with second year OSCE and undergraduate grade point average [26]. We speculate that their work supports our result. We suggest that as in the JRMP selection, the employers may evaluate the applicant on attitudes such as cooperativeness, honesty, and positivity as well as the score itself.

### Limitations

This research has some limitations; one is that the quantitative limit of the number of participants. The participants were 237 students of The UTokyo. We need more participants to increase the importance and validity of this study. Second, this study has been undertaken only in one institute. We are now planning to widen this study to multiple institutions.

Another limitation is that this study only investigated the scores of grades fourth-, fifth-, and sixth-year medical students. To raise the certainty of the whole study, we need to widen this study to lower and higher ages. In Japan the common evaluation system of medical competency throughout, before and after graduation of medical school is now being developed (EPOC2) [27]. In the near future EPOC2 may serve as one of the evaluation methods of medical competency.

## Conclusions

Our results show that the result of high-stakes examination before the CC, the pre-CC OSCE has a significant relationship with JRMP, the residency matching program in Japan. In order to achieve good educational outcomes such as high JRMP matching rates as well as NBE results, we should focus on pre-CC OSCE results and put more educational resources on those who did not achieve good scores on their pre-CC OSCE, especially in the medical interview and neurological examination stations.

## Data Availability

All data are kept in an available format in a secure cloud provided by The UTokyo.
